# Enhanced hair regrowth with five monthly sessions of minoxidil-dutasteride-copper peptides tattooing for androgenetic alopecia assessed by artificial intelligence and blinded evaluators

**DOI:** 10.1016/j.jdin.2025.01.012

**Published:** 2025-02-27

**Authors:** Guilherme Kuceki, Austin J. Coppinger, Sara D. Ragi, Luke S. Johnson, Andy Goren, Luiza L. Kalil, Pablo Cirino, Carlos G. Wambier

**Affiliations:** a Department of Dermatology, Ohio Health Riverside, Columbus, Ohio; b Department of Dermatology, The Warren Alpert Medical School of Brown University, Providence, Rhode Island; c Department of Dermatology, University of Utah Health, Salt Lake City, Utah; d Department of Dermatology, University of Rome G. Marconi, Rome, Italy; e Department of Dermatology, Yale School of Medicine, New Haven, Connecticut; f Department of Dermatology, University of São Paulo School of Medicine, São Paulo, Brazil

**Keywords:** alopecia, androgenetic alopecia, antiandrogens, baldness, drug delivery, dutasteride, hair loss, male pattern hair loss, microinfusion, minoxidil, microneedling, tattoo machine

*To the Editor:* Systemic, topical, and local injections of dutasteride and minoxidil have been established as effective treatments for androgenetic alopecia.^1^^,^^2^ Additionally, copper peptides have exhibited a stimulatory effect on hair follicle growth.^3^ A novel approach involving dermal infusion of these medications via scalp tattooing has demonstrated both safety and efficacy as a therapeutic option. However, existing studies have been limited to 3 monthly treatment sessions, and the impact of increasing the number of sessions has not yet been examined.^4^^,^^5^ The aim of our study was to evaluate the outcomes of 5 monthly sessions of minoxidil-dutasteride-copper peptide tattooing (MDCT).

We conducted a retrospective analysis of male patients who underwent MDCT between 2018 and 2019, following an unsatisfactory response to at least 1 year of standard oral and topical treatments. Seven male patients who completed 5 MDCT sessions were included in the study. Deidentified and randomized photographs, taken at baseline and 1 month posttreatment, were subjected to blinded evaluation using the top-quadrant Severity of Alopecia Tool (SALT) by 4 dermatologists via an online survey. We also employed ChatGPT's (version 4o) visual estimation capabilities to assess scalp visibility for each image. This complementary analysis was conducted to provide a standardized evaluation in the absence of trichoscopy images. The primary outcome was defined as achieving >10% top scalp area regrowth (TSAR), calculated as the difference between baseline and posttreatment top-quadrant SALT scores.

After cleansing of the scalp, a rotary tattoo machine (Cheyenne, MT.DERM GmbH) utilizing a 27-needle cartridge oscillating at 70 Hz (1890 perforations per second) with a 2-mm needle exposure to deliver a sterile compounded solution of 0.5% minoxidil sulfate (1 mL), 0.1% dutasteride (1 mL), and 1.2% copper peptides (1 mL). Analgesia was achieved through scalp lidocaine injections. Patients were instructed to continue using topical minoxidil, and none utilized oral finasteride, dutasteride, or minoxidil. The subjects were aged 28-55 years (median 42 years), with androgenetic alopecia severity classified as type III to IV on the Norwood-Hamilton scale. The median SALT scores were calculated for each photo. The median baseline top-quadrant SALT score was 40.0% (interquartile range: 23.8% to 50.0%), which reduced to 7.5% (interquartile range: 0% to 17.5%) at 5 months (Wilcoxon signed-rank test, *P* < .001) (Fig.1). Five patients (71.4%) achieved a median TSAR >10%. The median TSAR was 26.5% (interquartile range: 15.3% to 32.8%). ChatGPT's visual estimation achieved TSAR within 7.5% of the dermatologists' evaluations demonstrating close alignment with expert assessments, Table I.

**Fig 1:**
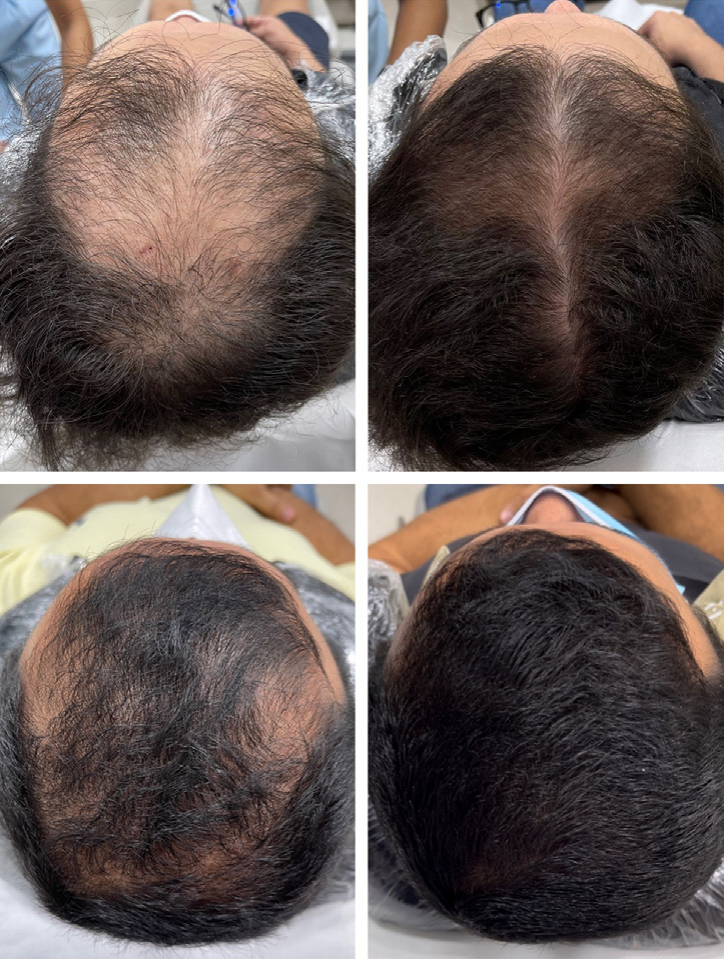
Androgenetic alopecia. Top scalp photographs with similar hair length. Baseline, the top Severity of Alopecia Tool median score was 50% and 30% respectively (upper photographs). After 6 months, the top Severity of Alopecia Tool score was 18.5% and 0% respectively (lower photographs). A median 35.5% and a 30% top scalp area regrowth were observed after 5 sessions of minoxidil, dutasteride, and copper peptides dermal drug delivery through tattooing.

**Table I:** Comparison of the median dermatologist SALT scores with ChatGPT's visual estimation of scalp visibility

Image	ChatGPT scalp visibility (%)	Dermatologist median SALT score	ChatGPT TSAR	Dermatologist median TSAR	Dermatologist mean TSAR
1A	60% to 70%	50	40	35.5	39
1B	20% to 30%	18.5			
2A	50% to 60%	29	20	26.5	22
2B	30% to 40%	10			
3A	60% to 70%	57.5	50	57.5	53.25
3B	10% to 20%	0			
4A	40%-50%	30	30	30	30.75
4B	10% to 20%	0			
5A	30% to 40%	10	10	4.5	4.75
5B	20% to 30%	6.5			
6A	50% to 60%	52.5	0	7.5	6.25
6B	50% to 60%	50			
7A	40% to 50%	30	20	23	25.5
7B	20% to 30%	7			

For simplicity, photos were labeled using the patient number, with “A” indicating pre-MDCT and “B” denoting 1 month posttreatment completion.

*SALT*, Severity of Alopecia Tool; *TSAR*, top scalp area regrowth.

When compared to the previously studied 3-monthly sessions of minoxidil-dutasteride tattooing (MDT),^4^ 5-monthly sessions of MDCT demonstrated an increased median TSAR (10% vs 26.5%, *P* = .0025, Mann-Whitney U test). Key differences include the increased number of sessions, the incorporation of copper peptides, and greater needle exposure (2 mm instead of 1.5 mm). No adverse reactions, such as scarring or infection, were observed in the subjects.

Limitations include the absence of objective scoring measures, such as trichoscopic hair density evaluations and the lack of a control arm.

The MDCT procedure, when administered as a five-session protocol, appears to be preliminarily effective in treating androgenetic alopecia. Future studies are warranted.

## Conflicts of interest

Dr Wambier has served as an adviser for Chemistry Rx, Daniel Alain, and Young Pharmaceuticals and as an investigator for Incyte, Eli Lilly, Pfizer, SunPharma, and UCB. Dr Kuceki, Coppinger, Ragi, Dr Johnson, Dr Goren, Dr Kalil, and Dr Cirino have no conflicts of interest to declare.
